# The Impact of Gamification-Induced Users' Feelings on the Continued Use of mHealth Apps: A Structural Equation Model With the Self-Determination Theory Approach

**DOI:** 10.2196/24546

**Published:** 2021-08-12

**Authors:** Tong Wang, Lingye Fan, Xu Zheng, Wei Wang, Jun Liang, Kai An, Mei Ju, Jianbo Lei

**Affiliations:** 1 Department of Medical Informatics, School of Public Health Jilin University Changchun China; 2 Department of Obstetrics and Gynecology, Affiliated Hospital Southwest Medical University Luzhou China; 3 Peking University Third Hospital Beijing China; 4 IT Center, Second Affiliated Hospital, School of Medicine Zhejiang University Hangzhou China; 5 School of Nursing Southwest Medical University Luzhou China; 6 Institute of Medical Technology, Health Science Center Peking University Beijing China; 7 Center for Medical Informatics, Health Science Center Peking University Beijing China; 8 School of Medical Informatics and Engineering Southwest Medical University Luzhou China

**Keywords:** mHealth app, continued use, continuance intention, gamification, self-determination theory (SDT), expectation confirmation model of information system continuance (ECM-ISC), PLS-SEM

## Abstract

**Background:**

Continued use of mHealth apps can achieve better effects in health management. Gamification is an important factor in promoting users’ intention to continue using mHealth apps. Past research has rarely explored the factors underlying the continued use of mobile health (mHealth) apps and gamification’s impact mechanism or path on continued use.

**Objective:**

This study aimed to explore the factors influencing mHealth app users’ intention to continue using mHealth apps and the impact mechanism and path of users’ feelings induced by gamification on continued mHealth app use.

**Methods:**

First, based on the expectation confirmation model of information system continuance, we built a theoretical model for continued use of mHealth apps based on users’ feelings toward gamification. We used self-determination theory to analyze gamification’s impact on user perceptions and set the resulting feelings (competence, autonomy, and relatedness) as constructs in the model. Second, we used the survey method to validate the research model, and we used partial least squares to analyze the data.

**Results:**

A total of 2988 responses were collected from mHealth app users, and 307 responses were included in the structural equation model after passing the acceptance criteria. The intrinsic motivation for using mHealth apps is significantly affected by autonomy (β=.312; *P*<.001), competence (β=.346; *P*<.001), and relatedness (β=.165; *P*=.004) induced by gamification. The intrinsic motivation for using mHealth apps has a significant impact on satisfaction (β=.311, *P*<.001) and continuance intention (β=.142; *P*=.045); furthermore, satisfaction impacts continuance intention significantly (β=.415; *P*<.001). Confirmation has a significant impact on perceived usefulness (β=.859; *P*<.001) and satisfaction (β=.391; *P*<.001), and perceived usefulness has a significant impact on satisfaction (β=.269; *P*<.001) and continuance intention (β=.273; *P*=.001). The mediating effect analysis showed that in the impact path of the intrinsic motivation for using the mHealth apps on continuance intention, satisfaction plays a partial mediating role (β=.129; *P*<.001), with a variance accounted for of 0.466.

**Conclusions:**

This study explored the impact path of users’ feelings induced by gamification on the intention of continued mHealth app use. We confirmed that perceived usefulness, confirmation, and satisfaction in the classical continued use theory for nonmedical information systems positively affect continuance intention. We also found that the path and mechanism of users' feelings regarding autonomy, competence, and relatedness generated during interactions with different gamification elements promote the continued use of mHealth apps.

## Introduction

### Background

Global health continues to face significant challenges, and health management is an important research direction. In 2016, 41 million people died of noncommunicable diseases globally, equivalent to 71% of global deaths [1]. One study showed that after 1 year of health management intervention, subjects’ risks of hypertension and hyperglycemia were reduced by 42.78% and 31.13%, respectively [2]. Moreover, every dollar invested in health management can reduce the cost of medical care by US $1 to $3 [3]. With mobile health (mHealth) technology maturity, the traditional health management model has gradually shifted to a management model based on mobile health.

With the popularization of smartphones, mHealth centered around mHealth apps has become an important means of health management. Related research is also increasing every year, mainly focusing on the health management effect, framework design, and user behavior regarding mHealth apps. According to estimates, more than 250,000 mHealth apps were on the market in 2016. It is estimated that there will be 4.68 billion mobile phone users worldwide in 2019, and the number of mHealth apps will achieve an annual growth rate of 41% between 2015 and 2020 [4]. mHealth can provide users with health intervention [5] and effective health evaluation indicators [6] and improve the communication between patients and health care professionals [7]. The number of studies on mHealth apps is also increasing every year. As of July 6, 2020, there were 688 articles related to mHealth apps in PubMed. Most studies have explored the intervention effect of mHealth apps [8-11]. Some studies focus on the development and design of mHealth apps to increase personalization and efficiency in health management services [12]. A few studies analyzed the acceptance and continued use of mHealth apps [13,14].

The effectiveness of health management based on an mHealth app is affected by user behavior. Short-term use can hardly achieve the expected goal of health management. However, the continued use of an mHealth app is not optimistic; most users only use it 4 times, and 25% use it only once after installation [14]. Therefore, it is essential to explore the influencing factors on users’ continued use of mHealth apps for the sustainable development of mHealth. Previous studies have explored the factors influencing the continued use of different types of apps. The technology acceptance model, expectation confirmation theory, unified theory of acceptance and use of technology, and other theories have been used to explore the influencing factors of continued app use, such as mobile instant messaging [15], mobile social apps [16], mobile social tourism and shopping [17], and paid mobile apps [18]. Compared to other apps, mHealth apps have different application scenarios and purposes, so it is unknown whether the research conclusions in other fields apply to mHealth apps. Therefore, some studies have explored the influencing factors of the continued mHealth app use from the aspects of quality, usability, user perception, and social impact. With the increasing number of new elements incorporated into applications, gamification is widely used in mHealth apps because it is believed to have the potential to promote continued use [19]. Out of 1000 mHealth apps, 772 contain at least 1 gamification element [20]. However, the effect of gamification in different apps is diverse [21], so whether gamification in mHealth apps has a positive impact on continued use needs to be explored.

Studies on the influence of gamification on mHealth apps’ continued use are limited. Previous studies have shown that gamification has a significant impact on the intention to continue using other types of apps [20,22,23], but whether it has the same effect on mHealth apps needs to be verified. Therefore, studies are gradually trying to explain the impact of gamification on behavioral intention in mHealth apps. For example, based on motivational feedback, some studies analyzed the factors influencing the continued use of fitness apps—an important type of mHealth app in terms of effective, informational, and social feedback—and proved the effect of gamification on affective feedback [22]. Other studies analyzed the effects of utilitarian benefits, hedonic benefits, and social benefits of gamified fitness apps on users' continuance intention and expanded the analysis of multiple impacts of social benefits on users' psychology and cognition [24]. Although previous research has substantially increased the understanding of the relationship between gamification and continued use, there are some limitations. Gamification does not cause all individuals to have the same subjective feelings and encourage them to continue using mHealth apps [25]. There is less discussion about the impact of different types of gamification on users' diverse feelings, and the resulting explanation of the different effects of gamification is insufficient. Therefore, we propose the following research questions:

1. What are the impact factors on continuance intention for gamified mHealth apps? What is the relationship between the impact factors?
2. By what path does users’ feeling induced by gamification affect users’ continuance intention toward mHealth apps?

To answer these questions, we built a research model to explain how users’ feelings induced by gamification affect the continuance intention for mHealth apps based on the expectation confirmation model of information system continuance (ECM-ISC) and self-determination theory (SDT). Specifically, SDT is used to analyze the impact of different types of gamification elements on users' feelings, and different feelings are set as constructs added to the model to explore the impact mechanism and path of gamification on the continued mHealth app use.

### Significance of This Study

Based on the ECM-ISC model, we introduced SDT theory and innovatively used this composite model to explain psychological aspects of gamification elements that affect the motivation to use mHealth apps to promote the continuance intention of mHealth app use. Previous studies on continuance mainly focused on information systems such as hospital information systems and massive open online courses that focused on improving efficiency. There is insufficient research on the factors influencing the continued use induced by mHealth app gamification. Second, we linked gamification with the continued use model through SDT and added the users’ feelings caused by different gamification elements into the research model. This paper proposes a model to explain the impact of users’ feelings toward gamification on continued mHealth app use and explores the impact mechanism and path of gamification, an emerging information technology method combined with behavior change theory, on the continued mHealth app use.

We first conducted an empirical test on the continuance model of the gamified mHealth app and, for the first time, verified the applicability of the classic model ECM-ISC in the mHealth app field for Chinese users. Compared with other studies on continuance, we did not choose a specific app for verification and included more apps to increase the applicability of the results. Second, we analyzed the impact of users’ feelings induced by gamification on promoting continued mHealth app use and provided a reference for mHealth app designers and researchers to promote users’ continuance intention for the mHealth app and improve the effect of users’ health management.

### Research Model and Hypotheses

**Figure 1:**
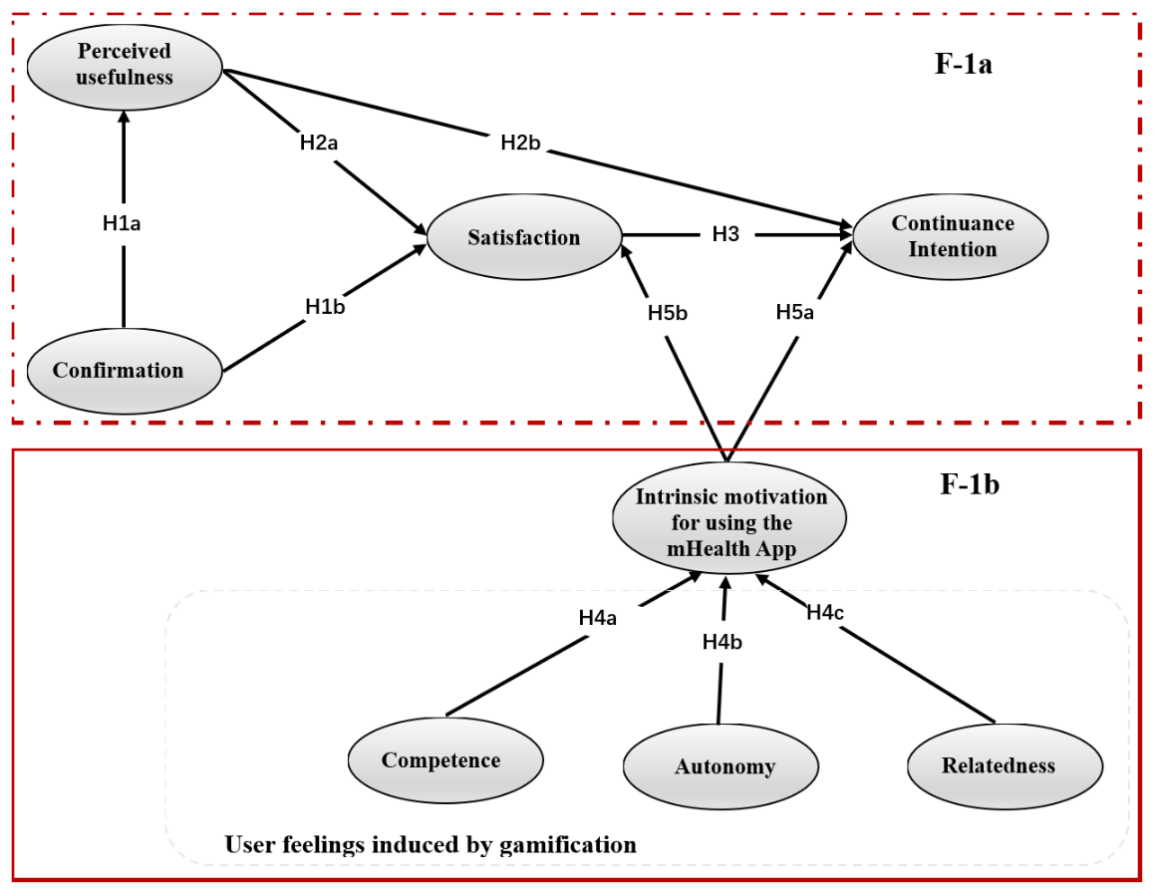
Research model.

Based on the ECM-ISC, combined with SDT, we explored the impact factors of the continued use of the gamified mHealth app. The research model and assumptions are shown in Figure 1. F-1a aims to verify the applicability of ECM-ISC in the mHealth app, and F-1b explores the impact factors of feelings induced by gamification based on SDT.

#### Hypotheses in ECM-ISC

The ECM-ISC (Figure 1, see F-1a) explains the process by which users generate continuance intention. Referring to the study by Bhattacherjee [26], the definitions of each construct are as follows: (1) confirmation is defined as users' perception of the congruence between expectations of mHealth apps use and its actual performance, (2) perceived usefulness refers to users' perception of the expected benefits of mHealth app use, (3) satisfaction is users' affect (feelings about) regarding prior mHealth apps use, (4) continuance intention is the user's willingness and tendency to continue using the mHealth app. Previous studies have revealed the relationship between these factors in nonmedical information systems [26]. Therefore, we believe that the same following assumptions exist in the mHealth app:

1. H1a: Confirmation positively influences perceived usefulness.
2. H1b: Confirmation positively influences satisfaction.
3. H2a: Perceived usefulness influences satisfaction.
4. H2b: Perceived usefulness influences continuance intention.
5. H3: Satisfaction influences continuance intention.

#### Hypothesis of Constructs Generated by Gamification Elements

SDT is a theory about human motivation and personality that highlights the importance of human-evolved inner resources for personality development and behavioral self-regulation. SDT defines 3 innate psychological needs: the needs for competence, autonomy, and relatedness [27]. SDT asserts that external things allow users to have autonomy, competence, and relatedness by satisfying innate psychological needs. The 3 feelings increase humans’ intrinsic motivation [28]. Gamification is the application of game design elements in nongame contexts, so the essence of gamification in mHealth app is a design method of app. The gamification element is its manifestation, and it is objective within the app. The direct stimulus of behavior motivation is the user's personality and feelings rather than external factors. Therefore, we need to explore the effect of gamification on users' feelings and then learn its impacts on behavior motivation. To achieve this goal, we chose 5 of the most common gamification elements as research objects. According to the characteristics of the different elements, this paper analyzed the impact of gamification on users' feelings. In the context of this research, autonomy refers to gamification providing users with the right to choose and set specific gamification contents independently and to determine their health management content and set goals; therefore, users have autonomy over their behavior [28]. Health management is the primary goal of the mHealth app, so we mainly explore the user's right to choose and self-controlled behaviors in health management goals.

The common achievement or progression-oriented gamification elements include points, medals, and leaderboards (PML) [21]. Therefore, we assume that the user's competence will be affected after experiencing the above gamification elements. In the context of this article, competence means that users obtain gamified feedback after completing app health management tasks, and this feedback allows uses to understand their health management capabilities. Furthermore, social networking is a social-oriented gamification element, which can leverage the functions of friending, commenting, and sharing experiences [21]. In mHealth apps, the above functions are generally utilized by the health community. This process reduces a sense of isolation from others and satisfies user needs for relatedness, making users feel relatedness [28]. Furthermore, according to SDT, competence, autonomy, and relatedness can increase the intrinsic motivation to use the mHealth app, described as follows:

1. H4a: Competence induced by gamification positively influences intrinsic motivation for using the mHealth app.
2. H4b: Autonomy induced by gamification positively influences intrinsic motivation for using the mHealth app.
3. H4c: Relatedness induced by gamification positively influences intrinsic motivation for using the mHealth app.

#### Relationship Between Intrinsic Motivation for Using the mHealth app and Satisfaction and Continuance Intention

Intrinsic motivation drives human behaviors, emphasizing that humans carry out certain activities based on their personalities and feelings [27]. Feelings are not easy to change, so intrinsic motivation is persistent; therefore, the intrinsic motivations of the mHealth app promote continuance intention. Under the influence of their feelings and interests, users will actively use the mHealth app, resulting in satisfactory evaluations. Therefore, we hypothesize the following:

1. H5a: Intrinsic motivation for using the mHealth app positively influences continuance intention.
2. H5b: Intrinsic motivation for using the mHealth app positively influences satisfaction.

## Methods

### Measurement Instrument

This study constructed a new theoretical model to explore how users’ feelings induced by gamification promote the continued use of mHealth apps. It was necessary to select appropriate observable variables for the constructs to verify our model. To develop the measurement instrument of the survey, we adapted previously validated scales to our research context. To design the measurement instrument reasonably, we adjusted the previously validated scales according to the experimental purpose. For the feelings caused by gamification elements, items for competence were drawn from McAuley et al [29]. Items for autonomy were sourced from several studies [29-31]. Items for relatedness were from 3 studies [30-32]. Items for intrinsic motivation for using mHealth apps were from Lin [33]. Scales for ECM-ISC, items for perceived usefulness, satisfaction, confirmation, and continuance intention were drawn from validated scales [26,34,35]. We used the 7-point Likert scale to measure the items with anchors ranging from 1 (strongly disagree) to 7 (strongly agree). We used the initial questionnaire to presurvey 17 testers and modified the scale based on evaluations and suggestions to form the final questionnaire (see Multimedia Appendix 1). We used the data collection service from China's largest online survey platform to manage the survey [36].

### Research Target and Data Collection

**Figure 2:**
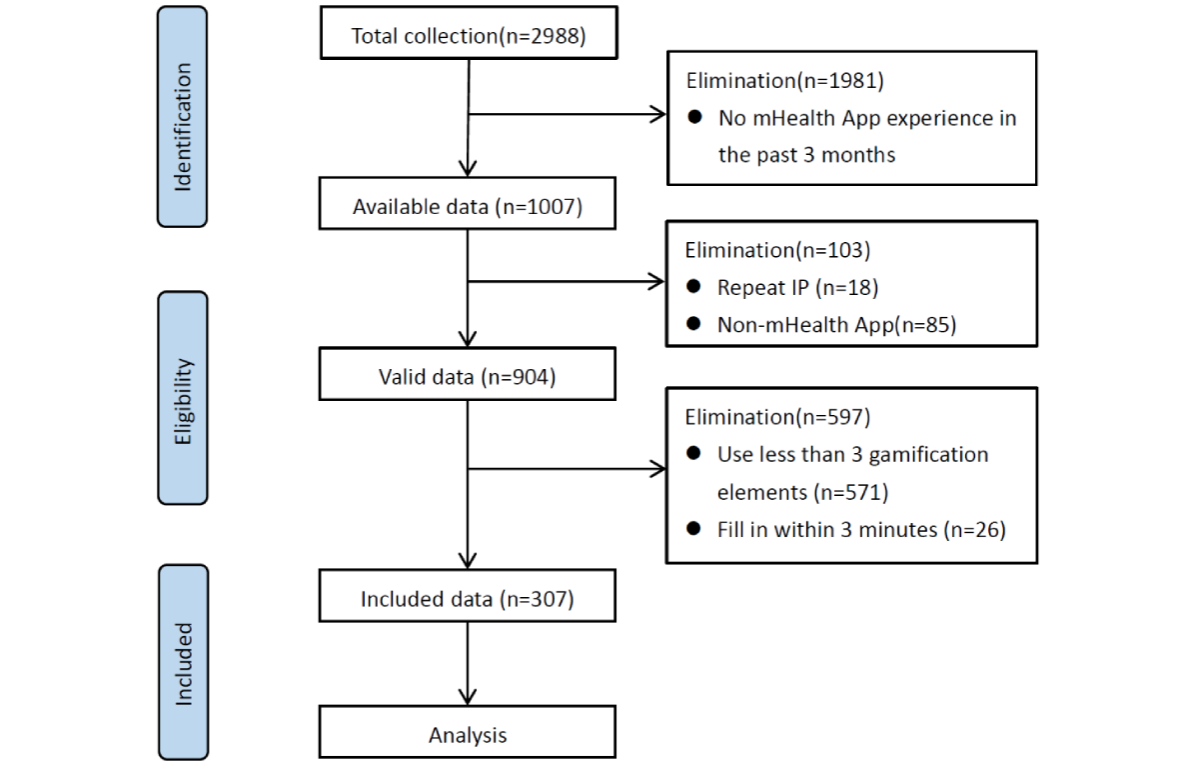
Data inclusion and exclusion process. IP: internet protocol; mHealth: mobile health.

The study subjects have used an mHealth app in the past 3 months. From January 20 to February 23, 2020, we used WeChat to conduct a snowball sampling survey, posting the link to the questionnaire on the WeChat moments and WeChat group. A total of 2988 questionnaires were returned, mainly distributed across 29 regions of China. In addition, 11 responses from Chinese users were also received from the United States, Australia, and Austria. We excluded responses that included the following characteristics: no mHealth app experience in the past 3 months (n=1981, 66.30%), repeat internet protocols (IP) (n=18, 0.60%), non-mHealth app (n=85, 2.84%), use of fewer than 3 gamification elements (n=571, 19.11%), and completion within 3 minutes (n=26, 0.87%). Finally, we obtained a valid questionnaire (n=307, 10.27%). No mHealth app experience means that users replied that they had not used the mHealth app in the past 3 months. A non-mHealth app means the app used by the user was not a mHealth app. We determined whether the app is an mHealth app according to the classification system of Apple’s App Store. If the app used by the user was not included in the “health and fitness” and “medical” categories, it was regarded as a “non-mHealth app.” The data inclusion and exclusion process are shown in Figure 2. In addition, although we selected users who have used at least 3 gamification elements, some users still needed to answer a few items that include a gamification element that users did not use. For the items involving in the gamification elements that participants did not use, we input point 4 (uncertainty) on the 7-point Likert scale. This study was approved by the Biomedical Ethics Committee of Peking University, and the subjects were aware of the study’s purpose and process.

### Data Analysis

The partial least squares structural equation model (PLS-SEM) is more suitable for exploratory research than the covariance-based structural equation model [37]. PLS-SEM can deal with non-normal data and complex models with more latent variables [38] and has lower sample requirements [10]. Our research is exploratory, and the data are not strictly normally distributed; therefore, this study uses PLS-SEM analysis with SmartPLS (version 3.2.8; SmartPLS GmbH).

## Results

### Demographic Information

The demographic information for the included samples is shown in Table 1. We divided the apps into 4 categories according to their functions, including menstruation and pregnancy management, fitness and diet management, online consultation, etc. We also investigated the users' health status during follow-up to explore whether health status has a regulatory effect on continued use. The users’ health was indirectly measured by perceived health using a 7-point Likert scale from 1 (very unhealthy) to 7 (very healthy), with an average of 5.35 (SD 1.193).

**Table 1:** Demographic information.

Characteristics		Users, n (%)
**Gender**		
	Male	174 (56.7)
	Female	133 (43.3
**Age**		
	≤30	101 (32.9)
	31-45	132 (43.0)
	46-60	67 (21.8)
	≥61	7 (2.3)
**Education**		
	High school	4 (1.3)
	Junior college	22 (7.2)
	College	130 (42.3)
	Master’s degree and above	151 (49.2)
**Income**		
	≤2500	60 (19.5)
	2501-5000	28 (9.1)
	5001-8000	57 (18.6)
	8001-30,000	125 (40.7)
	≥30,001	37 (12.1)
**Frequency**		
	≤1 time per week	83 (27.0)
	2-3 times per week	95 (30.9)
	4-5 times per week	58 (18.9)
	6-7 times per week	45 (14.7)
	>7 times per week	26 (8.5)
**Occupation**		
	Students	58 (18.9)
	Medicine-related personnel	70 (22.8)
	Public servants and clerks	77 (25.1)
	Commercial and service personnel	28 (9.1)
	Professional technical personnel	66 (21.5)
	Manual workers	4 (1.3)
	Unemployed and other personnel	4 (1.3)
**Type of mHealth app**		
	Menstruation and pregnancy management	11 (3.6)
	Fitness and diet management	212 (69.1)
	Online consultation	37 (12.1)
	Others	47 (15.3)

### Analysis Result of Hypothesized Model

#### Measurement Model

The reliability and validity of the questionnaire were measured by confirmatory factor analysis. The results are shown in Table 2. To test the model’s reliability, we calculated Cronbach’s alpha and composite reliability based on the score of each item. A value of 0.5 represents acceptable reliability, and 0.7 represents good reliability [39]. To verify the convergent validity, we calculated the project load and the value of average extracted variance (AVE). The larger the factor loading, the greater the influence of the observed variable on the latent variable. The commonly adopted acceptance standard is 0.7. AVE represents the degree of aggregation of different items in the same construct, and a value greater than 0.5 represents acceptable convergence validity [39]. As shown in Table 2, all the values of Cronbach’s alpha and composite reliabilities are above 0.8, the AVE of each construct is above 0.6, and the loading weights for each item are above 0.7, indicating good reliability for all constructs and good convergent validity. As shown in Table 3, we compare the correlation between the AVE square root of each construct and other constructs to show that the observed variable of the same construct is not related to other latent variables. The results show that the square root of AVE of each structure is greater than the interconstruct correlations, showing good discriminant validity [40]. Therefore, we conclude that the results of the measurement model are sufficient to test the hypotheses in the model.

Considering that all constructs are measured by a scale, common method bias needs to be tested to eliminate systematic errors caused by the same data source and measurement environment. We performed a statistical analysis that is suitable for the PLS-SEM model. We added a common factor to the model, which contains all items, and calculated each indicator's substantive variances explained by the principal construct and by the method [41]. The average substantive variance of the indicators is 0.904, while the average method-based variance is 0.007. The ratio of substantive variance to method variance is approximately 129:1 (see Multimedia Appendix 2). Given the small magnitude and insignificance of method variance, we believe that there is no serious common method bias in this study. The multicollinearity was checked by calculating the variance inflation factor (VIF) [42]. The VIF values of all the regressions were all substantially below the cutoff value of 10, which means there are no severe multicollinearity problems (see Multimedia Appendix 2).

**Table 2:** Construct reliability and convergent validity.

Construct and items		Factor loadings	Composite reliability	Average variance extracted	Cronbach alphas
**Autonomy (AUT)**					
	AUT1	0.908	0.935	0.827	0.896
	AUT2	0.914			
	AUT3	0.906			
**Competence (COMP)**					
	COMP1	0.806	0.891	0.6732	0.817
	COMP2	0.874			
	COMP3	0.883			
**Relatedness (REL)**					
	REL1	0.847	0.936	0.786	0.909
	REL2	0.864			
	REL3	0.925			
	REL4	0.907			
**Continuance intention (CI)**					
	CI1	0.888	0.925	0.754	0.891
	CI2	0.799			
	CI3	0.884			
	CI4	0.900			
**Confirmation (CONF)**					
	CONF1	0.943	0.967	0.906	0.948
	CONF2	0.968			
	CONF3	0.945			
**Intrinsic motivation for using the mHealth app (MOT)**					
	MOT1	0.914	0.949	0.823	0.928
	MOT2	0.926			
	MOT3	0.926			
	MOT4	0.862			
**Perceived usefulness (USE)**					
	USE1	0.931	0.961	0.892	0.940
	USE2	0.961			
	USE3	0.941			
**Satisfaction (SAT)**					
	SAT1	0.932	0.949	0.861	0.919
	SAT2	0.916			
	SAT3	0.937			

**Table 3:** Discriminant validity.

	AUT^a^	COMP^b^	CONF^c^	CI^d^	MOT^e^	USE^f^	REL^g^	SAT^h^
AUT	*0.909* ^i^							
COMP	0.355	*0.855*						
CONF	0.445	0.490	*0.952*					
CI	0.427	0.458	0.779	*0.869*				
MOT	0.464	0.546	0.760	0.710	*0.907*			
USE	0.505	0.545	0.859	0.754	0.785	*0.945*		
REL	0.182	0.547	0.352	0.310	0.411	0.362	*0.886*	
SAT	0.512	0.507	0.859	0.781	0.820	0.849	0.373	*0.928*

^a^AUT: autonomy.

^b^COMP: competence.

^c^CONF: confirmation.

^d^CI: continuance intention.

^e^MOT: intrinsic motivation for using the mHealth app.

^f^USE: perceived usefulness.

^g^REL: relatedness.

^h^SAT: satisfaction.

^i^Italics refers to the square roots of average variance extracted.

#### Structural Model

**Figure 3:**
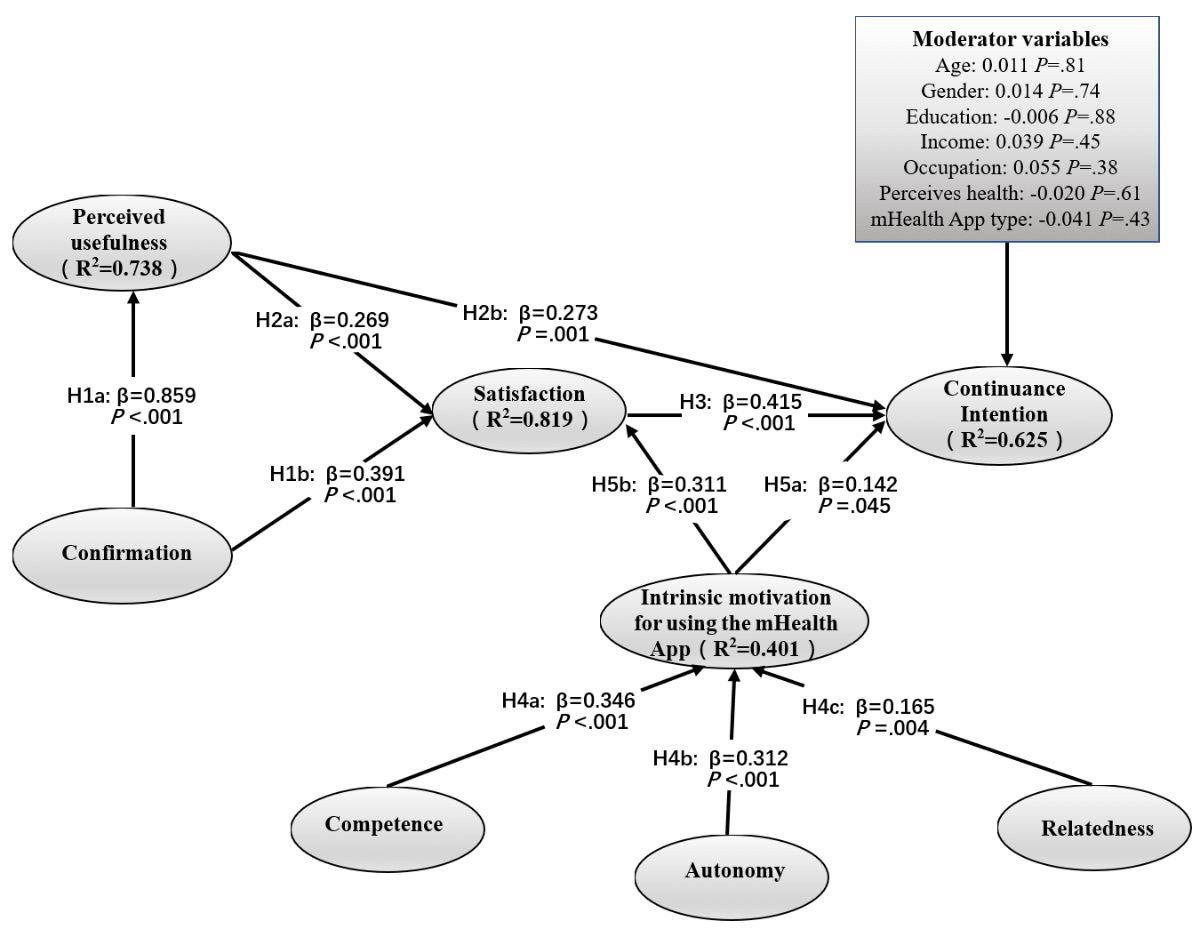
Analysis results of structural model. mHealth: mobile health.

Through the bootstrapping analysis in PLS-SEM [43], we tested the hypotheses in the research model. As shown in Figure 3, there is an explanation for variance R^2^ and standardized path coefficient β. R^2^ refers to the variance of endogenous latent variables that can be explained by exogenous latent variables. The exogenous latent variable explains the variance, and β represents the correlation between the variables. First, regarding the applicability of ECM-ISC in an mHealth app, confirmation significantly affected both perceived usefulness and satisfaction, and perceived usefulness significantly affected satisfaction and continuance intention for the mHealth app. In addition, satisfaction significantly impacted the continuance intention for the mHealth app. Therefore, H1a, H1b, H2a, H2b, and H3 were supported. The above results tested the applicability of ECM-ISC in the mHealth app. Second, the constructs related to gamification, competence, autonomy, and relatedness significantly influenced the intrinsic motivation for using an mHealth app. Therefore, H4a, H4b, and H4c were all supported. Intrinsic motivation for using an mHealth app significantly impacted satisfaction and continuance intention for the mHealth app; thus, H5a and H5b were supported. As shown in Figure 3, age, gender, education, income, occupation, and other social demographic characteristics have no significant moderating effects on the results.

### Results of Mediation Analysis

PLS-SEM and bootstrapping analysis results included direct effects, indirect effects, and total effects between variables. These allowed us to directly use variance accounted for (VAF) to analyze the mediating effects between variables. VAF measures the ratio of indirect effects to total effects. A VAF greater than 0.8 is generally considered a complete mediation effect, and a partial mediation effect is between 0.2 and 0.8 [44]. As shown in Table 4, all the effects were significant, whereas satisfaction was a partial mediator variable between motivation for using an mHealth app and continuance intention for the mHealth app and between perceived usefulness and continuance intention for the mHealth app. In addition, perceived usefulness was a partial mediator variable between confirmation and satisfaction.

**Table 4:** Mediation analysis.

Independent variable	Mediating variable	Dependent variable	Indirect effects		Total effects		VAF^a^
			β	*P* value	β	*P* value	
Intrinsic motivation	Satisfaction	Continuance	.129	<.001	.277	<.001	0.466
Usefulness	Satisfaction	Continuance	.112	.002	.386	<.001	0.290
Confirmation	Usefulness	Satisfaction	.231	<.001	.621	<.001	0.372

^a^VAR: variance accounted for.

## Discussion

### Most Current Gamification in the mHealth App Is Unattractive to Users

In the study, 1007 users had mHealth app experience in the past 3 months, but 248 (24.63%) users believed they had not used gamification elements. Auditing the questionnaires of the 248 users, we found that 109 (43.95%) of them used apps that contained at least 1 gamification element. Gamification has not attracted the users’ attention and interest because some gamification advocates believe that the golden combination of PML can work in any app [25], leading some designers to simply combine gamification elements applied in the mHealth app with no characteristics and without considering the users’ unique needs and intrinsic motivations. Although gamification elements are widely used in mHealth apps, the design process lacks an understanding of gamification and users’ feelings; therefore, they cannot attract users.

### The Impact Path of Gamification on Continuance Intention for mHealth Apps

This study shows that users’ autonomy, competence, and relatedness interacting with different gamification elements positively promote their motivation for using an mHealth app, followed by the continuance intention for the mHealth app. This path also explains the ineffectiveness of gamification on some users. For example, if users do not feel autonomy, competence, and relatedness in the process of interacting with gamification, then gamification will not have a significant impact on the continued use of an mHealth app for this type of user.

The explanation of the impact path is as follows. First, users hope to make personalized settings in the mHealth app, such as health management tasks and user image [45]. The personalized settings allow users to experience tailor-made services, and they cater to users’ interests. Autonomy is generated during the process of experiencing gamification elements such as optional goals, which increases intrinsic motivation for using the mHealth app. Second, social interaction within the mHealth app promotes the users’ intention for use, indicating that the more online friends and the more frequent the interactions between users, the more significant the impact of social interaction on users’ mHealth app use [46,47]. Users share health management experiences through online communities. In experiencing gamification elements with social orientation, there are interactive behaviors among users. These behaviors help users receive support and care and feel relatedness, increasing the intrinsic motivation for using the mHealth app. Finally, this study confirmed that competence caused by gamification promoted motivation for using the mHealth app. The representative gamification elements that affect competence in this study are PML. In mHealth apps, users need to complete health management tasks (eg, exercise, diet control, etc) to obtain such elements. These elements show users' progress in health management, enhancing their competence and increasing their motivation to use mHealth apps. However, it is difficult for users to change their health status after completing a health management task, and long-term and frequent use can achieve the desired effect [14]. Therefore, it is very important to reasonably design gamification elements to induce users’ sense of competence, relatedness, and autonomy, increasing users’ motivation to use mHealth apps.

### The Influence Mechanism of Gamification on the Continuance Intention of the mHealth App

In the continued use model of the gamified mHealth app, significant impacts include confirmation, perceived usefulness, satisfaction, motivation for using the mHealth app, autonomy, competence, relatedness, and continuance intention. Among them, satisfaction and motivation for using the mHealth app have direct effects on continuance intention. Satisfaction mediates the influence of motivation for using the mHealth app on continuance intention.

High intrinsic motivation means that users’ interests and feelings, which are not easily affected by external factors, are satisfied. Therefore, intrinsic motivation increases satisfaction. Furthermore, it is more likely that the user’s inner emotional motivation will continue to exist, so the intrinsic motivation for using the mHealth app promotes continuance intention. This is consistent with conclusions by Cruz et al [48] and and Chang [49]. The results of the mediating effect analysis showed that satisfaction is the mediating factor explaining continuance intention, which is consistent with the conclusions from Hsiao et al [16]. Therefore, we concluded that the motivation for using an mHealth app has both direct and indirect effects on mHealth app continuance intention.

### Limitations and Future Research

First, we collected data using snowball sampling, and the questionnaire was distributed through WeChat. Although the participants were distributed across the country, the sample was not enough to represent all mHealth app users, which may cause bias in the results.

Second, although we explored the impact of 5 common gamification elements on users' feelings, we did not analyze the specific role of each element and the differences between the impact of each gamification element on users' feelings. However, research in this direction is of great significance for understanding gamification. Therefore, our subsequent research focus will distinguish the effects of different types of gamification elements on users' feelings and continuance intention.

### Conclusion

Based on ECM-ISC and SDT, we established a theoretical model of gamified mHealth app continuance and validated the proposed research model. Specifically, this study not only verifies the applicability of ECM-ISC to mHealth apps but also confirms that perceived usefulness, confirmation, and satisfaction positively impact mHealth app continuance intention. In terms of the impact mechanism, the competence users experience in their interaction with achievement and progress-oriented gamification elements in mHealth application, the relatedness users experience during their interaction with social-oriented gamification elements, and the autonomy users experience in their interaction with personalized service gamification elements enhance the intrinsic motivation of using mHealth apps, thus promoting the continuance intention.

## Appendix

Multimedia Appendix 1 Measurement instrument.

Multimedia Appendix 2 CMV and Multicollinearity analysis.

Multimedia Appendix 3 Total collection’s demographic information.

## Abbreviations

AVE: average extracted variance
ECM-ISC: expectation confirmation model of information system continuance
IP: internet protocol
mHealth: mobile health
PLS-SEM: partial least squares structural equation model
PML: points, medals, and leaderboards
SDT: self-determination theory
VAF: variance accounted for
VIF: variance inflation factor
