# Supplementary material for: The Impact of Gamification-Induced Users' Feelings on the Continued Use of mHealth Apps: A Structural Equation Model With the Self-Determination Theory Approach
Source: J Med Internet Res. 2021 Aug 12;23(8):e24546. doi: 10.2196/24546 (PMC8391751; doi:10.2196/24546)
Supplement: Multimedia Appendix 1 [file jmir_v23i8e24546_app1.docx]

**Appendix 1 — Measurement instrument**

| Variables and sources | Original version | Formal Version | Chinese version |
| --- | --- | --- | --- |
| Continuance  ( Bhattacherjee, A. (2001), Rahi, S.（2019）) | - I intend to continue using OBD rather than discontinue its use. - My intentions are to continue using OBD than use any alternative means (traditional banking). - I plan to continue using internet banking frequently. - I intend to continue using internet banking in the future. | - 1. I intend to continue using the mHealth App I used rather than discontinue its use. - 2. My intentions are to continue using the mHealth App I used than use any alternative mHealth App. - 3. I plan to continue using the mHealth App I used frequently. - 4. I intend to continue the current frequency of using the mHealth App I used. | - 1. 我打算以后继续使用【这个健康app】，而不是停止使用。 - 2. 即使以后有别的替代软件，我也会继续使用当前【这个健康app】。 - 3. 我以后会尽可能多的使用【这个健康APP】。 - 4. 我以后会保持现在【这个健康app】的频率。 |
| Satisfaction  (Hsiao, J. L.（2019）, Rahi, S.（2019）) | - Overall, I am satisfied with the use of MH. - My experience with using MH is quite close to my ideal MHS. - I feel contented with using internet banking service. | - 1. Overall, I am satisfied with the use of mHealth App I used. - 2. My experience with using the mHealth App I used is quite close to my ideal mHealth App. - 3. I feel contented with using the mHealth App service I used. | - 1. 总的来说，我对【这个健康app】的使用感到满意。 - 2. 我使用的【这个健康app】非常接近我理想的健康管理软件。 - 3.我对【这个健康app】提供的服务感到满意。 |
| Usefulness  ( Rahi, S.（2019）) | - Using internet banking improves my working and living performance. - Using internet banking improves my working and living effectiveness. - Overall, internet banking website is useful. | - 1. Using the mHealth App I used improves my health managing performance. - 2. Using the mHealth App I used improves my health managing effectiveness. - 3. Overall, the mHealth App I used is useful. | - 1.【这个健康app】改善了我健康管理的能力。 - 2. 【这个健康app】提高了我健康管理的效率。 - 3. 【这个健康app】在健康管理方面是有用的。 |
| Confirmation ( Bhattacherjee, A. (2001)) | - My experience with using OBD was better than what I expected. - The service level provided by OBD was better than what I expected. - Overall, most of my expectations from using OBD were confirmed. | - 1. My experience with using the mHealth App I used was better than what I expected. - 2. The service level provided by the mHealth App I used was better than what I expected. - 3. Overall, most of my expectations from using the mHealth App I used were confirmed. | - 1. 【这个健康app】的使用感受比我预期的要好。 - 2. 【这个健康app】提供的功能和服务比我预期的要好。 - 3.总的来说，我对【这个健康app】的大多数预期都得到确认。 |
| Competence ( McAuley, E.（1989）) | - I am satisfied with my performance at this task. - After working at this activity for a while, I felt pretty competent. | - I gain more points in the mHealth App I used, which makes me feel more competent in health management. - I gain badges in the mHealth App I used, which makes me feel more competent in health management. - Progress on the leaderboard makes me feel more competent in health management. | - 1.我在【这个健康app】的积分升高让我感到自己健康管理能力提升。 - 2.我在【这个健康app】获得奖励勋章让我感到自己健康管理能力提升。 - 3. 在【这个健康app】排行榜上的进步让我感到自己健康管理能力提升。 |
| Autonomy ( McAuley, E.（1989）, La Guardia, J. G.（2000）, Inchamnan, W. (2016) ) | - The game provides me with interesting options and choices. - I believe I had some choice about doing this activity. - When I am with my__,I have a say in what happens and can voice my opinion. | - The mHealth App I used provides me with a variety of health management goals. - I believe I had some goals to choose about health management. - In the mHealth App I used, I can set my owe health management goals. | - 1.【这个健康app】为我提供了多种健康管理任务。 - 2.在【这个健康app】中我可以自主选择健康管理任务。 - 3.在【这个健康app】中我可以自己设定健康管理任务。 |
| Relatedness  ( Lee, Y.（2015）, Inchamnan, W. (2016), La Guardia, J. G.（2000）) | - The Qboard gives me more chances to interact with others. - I find the relationships I form in this game important. - When I am with my__,I often feel a lot of distance in our relationship.（R） | - The community in the mHealth App I used gives me more chances to interact with others. - I have interactive friends in the mHealth App I used, and this relationship is important to me. - When I share my health management experience in the mHealth App I used, I feel that the distance from other users is decreasing. - When I read other users’ health management experience in the mHealth App I used, I feel that the distance from other users is decreasing. | - 1.【这个健康app】的社区功能让我有更多机会与其他人互动。 - 2.在【这个健康app】中我有互动的好友，这个关系对我有意义。 - 3.在【这个健康app】分享健康管理经验时，我感到与其他用户的距离感在减少。 - 4.在【这个健康app】阅读他人的健康管理经验时，我感到与其他用户的距离感在减少。 |
| Motivation  (Lin, C. P.（2009）) | - Because I find this task interesting to do. - Because I like doing this task. - The service helps improve my skincare and appearance - I can apply what I learn from the salon in my daily life. | - I find the mHealth App I used interesting. - I like using the mHealth App I used. - The mHealth App I used helps me with health management. - I can apply what I learn from the mHealth App used in my daily life. | - 我认为【这个健康app】很有意思。 - 我喜欢使用【这个健康app】。 - 【这个健康app】有助于我进行健康管理。 - 我可以将【这个健康app】学到的知识用到日常生活中。 |
