# Supplementary material for: The Impact of Gamification-Induced Users' Feelings on the Continued Use of mHealth Apps: A Structural Equation Model With the Self-Determination Theory Approach
Source: J Med Internet Res. 2021 Aug 12;23(8):e24546. doi: 10.2196/24546 (PMC8391751; doi:10.2196/24546)
Supplement: Multimedia Appendix 2 [file jmir_v23i8e24546_app2.docx]

*Appendix 2*

*Common method bias analysis*

| *Construct* | *Indicator* | *Substantive Factor Loading(R1)* | *R1^2^* | *Method Factor Loading(R2)* | *R2^2^* |
| --- | --- | --- | --- | --- | --- |
| *autonomy* | *AUT1* | *0.891* | *0.794* | *0.117* | *0.014* |
|  | *AUT2* | *0.920* | *0.846* | *-0.049* | *0.002* |
|  | *AUT3* | *0.919* | *0.845* | *-0.066* | *0.004* |
| *continuance intention* | *CI1* | *0.884* | *0.781* | *0.074* | *0.005* |
|  | *CI2* | *0.808* | *0.653* | *-0.156* | *0.024* |
|  | *CI3* | *0.882* | *0.778* | *0.040* | *0.002* |
|  | *CI4* | *0.898* | *0.806* | *0.026* | *0.001* |
| *competence* | *COMP1* | *0.817* | *0.667* | *0.016* | *0.000* |
|  | *COMP2* | *0.888* | *0.789* | *-0.087* | *0.008* |
|  | *COMP3* | *0.862* | *0.743* | *0.072* | *0.005* |
| *confirmation* | *CONF1* | *0.944* | *0.891* | *-0.095* | *0.009* |
|  | *CONF2* | *0.968* | *0.937* | *-0.052* | *0.003* |
|  | *CONF3* | *0.943* | *0.889* | *0.146* | *0.021* |
| *intrinsic motivation for using the mHealth App* | *MOT1* | *0.913* | *0.834* | *0.072* | *0.005* |
|  | *MOT2* | *0.926* | *0.857* | *-0.001* | *0.000* |
|  | *MOT3* | *0.926* | *0.857* | *-0.013* | *0.000* |
|  | *MOT4* | *0.864* | *0.746* | *-0.063* | *0.004* |
| *relatedness* | *REL1* | *0.840* | *0.706* | *0.008* | *0.000* |
|  | *REL2* | *0.880* | *0.774* | *-0.067* | *0.004* |
|  | *REL3* | *0.926* | *0.857* | *-0.002* | *0.000* |
|  | *REL4* | *0.900* | *0.810* | *0.060* | *0.004* |
| *satisfaction* | *SAT1* | *0.931* | *0.867* | *0.011* | *0.000* |
|  | *SAT2* | *0.918* | *0.843* | *-0.078* | *0.006* |
|  | *SAT3* | *0.935* | *0.874* | *0.064* | *0.004* |
| *perceived usefulness* | *USE1* | *0.933* | *0.870* | *-0.198* | *0.039* |
|  | *USE2* | *0.961* | *0.924* | *0.033* | *0.001* |
|  | *USE3* | *0.940* | *0.884* | *0.157* | *0.025* |
| *average* |  | *0.904* | *0.819* | *-0.001* | *0.007* |

*Multicollinearity analysis*

| *model* | | *Multicollinearity analysis* | |
| --- | --- | --- | --- |
|  |  | *tolerance* | *VIF* |
|  | *(constant)* |  |  |
|  | *SAT* | *.181* | *5.538* |
|  | *CONF* | *.206* | *4.864* |
|  | *MOT* | *.290* | *3.454* |
|  | *COMP* | *.555* | *1.800* |
|  | *AUT* | *.710* | *1.408* |
|  | *REL* | *.687* | *1.456* |
|  | *USE* | *.198* | *5.046* |
| *Dependent Variable: CI* | | | |
