# Supplementary material for: The Impact of Gamification-Induced Users' Feelings on the Continued Use of mHealth Apps: A Structural Equation Model With the Self-Determination Theory Approach
Source: J Med Internet Res. 2021 Aug 12;23(8):e24546. doi: 10.2196/24546 (PMC8391751; doi:10.2196/24546)
Supplement: Multimedia Appendix 3 [file jmir_v23i8e24546_app3.docx]

*Appendix 3 — Total collection’s demographic information*

*Table 1. Total collection’s demographic information*

| *Characteristics* | | *Number* | *Percentage* |
| --- | --- | --- | --- |
| *Gender* | |  |  |
|  | *male* | *1480* | *49.53* |
|  | *female* | *1508* | *50.47* |
| *Age* | |  |  |
|  | *<=30* | *797* | *26.67* |
|  | *31~45* | *1083* | *36.24* |
|  | *46~60* | *1009* | *33.77* |
|  | *>=61* | *99* | *3.32* |
| *Education* | |  |  |
|  | *high school* | *317* | *10.61* |
|  | *junior college education* | *466* | *15.60* |
|  | *college* | *1120* | *37.48* |
|  | *master degree and above* | *1085* | *36.31* |
| *Income* | |  |  |
|  | *<=2500* | *645* | *21.59* |
|  | *2501~5000* | *479* | *16.03* |
|  | *5001~8000* | *534* | *17.87* |
|  | *8001~30000* | *1079* | *36.11* |
|  | *>=30001* | *251* | *8.40* |
| *Occupation* | |  |  |
|  | *students* | *433* | *14.49* |
|  | *medicine-related personnel* | *595* | *19.91* |
|  | *public servants and clerks* | *682* | *22.82* |
|  | *commercial and service personnel* | *366* | *12.25* |
|  | *professional technical personnel* | *558* | *18.67* |
|  | *manual workers* | *120* | *4.02* |
|  | *jobless and other personnel* | *234* | *7.83* |
